# Amino acid permease RcAAP1 increases the uptake and phloem translocation of an L-valine-phenazine-1-carboxylic acid conjugate

**DOI:** 10.3389/fpls.2023.1191250

**Published:** 2023-06-02

**Authors:** Yongxin Xiao, Ciyin Hu, Tom Hsiang, Junkai Li

**Affiliations:** ^1^ Hubei Key Laboratory of Waterlogging Disaster and Agricultural Use of Wetland, College of Agriculture, Yangtze University, Jingzhou, China; ^2^ School of Environmental Sciences, University of Guelph, Guelph, ON, Canada

**Keywords:** amino acid-pesticide conjugate, vacuum agroinfiltration, overexpression, subcellular localization, uptake, phloem mobility

## Abstract

Amino acid conjugates of pesticides can promote the phloem translocation of parent ingredients, allowing for the reduction of usage, and decreased environmental pollution. Plant transporters play important roles in the uptake and phloem translocation of such amino acid-pesticide conjugates such as L-Val-PCA (L-valine-phenazine-1-carboxylic acid conjugate). However, the effects of an amino acid permease, RcAAP1, on the uptake and phloem mobility of L-Val-PCA are still unclear. Here, the relative expression levels of *RcAAP1* were found to be up-regulated 2.7-fold and 2.2-fold by the qRT-PCR after L-Val-PCA treatments of *Ricinus* cotyledons for 1 h and 3 h, respectively. Subsequently, expression of *RcAAP1* in yeast cells increased the L-Val-PCA uptake (0.36 μmol/10^7^ cells), which was 2.1-fold higher than the control (0.17 μmol/10^7^ cells). Pfam analysis suggested RcAAP1 with its 11 transmembrane domains belongs to the amino acid transporter family. Phylogenetic analysis found RcAAP1 to be strongly similar to AAP3 in nine other species. Subcellular localization showed that fusion RcAAP1-eGFP proteins were observed in the plasma membrane of mesophyll cells and phloem cells. Furthermore, overexpression of *RcAAP1* for 72 h significantly increased the phloem mobility of L-Val-PCA in *Ricinus* seedlings, and phloem sap concentration of the conjugate was 1.8-fold higher than the control. Our study suggested that RcAAP1 as carrier was involved in the uptake and phloem translocation of L-Val-PCA, which could lay foundation for the utilization of amino acids and further development of vectorized agrochemicals.

## Introduction

The uptake and translocation of amino acids can occur in the phloem and is generally mediated by plasma membrane transporters (Bush, 1993; Fischer et al., 1998; Fischer et al., 2002). With transporters as carriers, vectorization of agrochemicals by conjugating amino acids to the parent ingredients can improve the phloem mobility, reduce usage, and thus decrease environmental pollution (Chollet et al., 1997; Wu et al., 2019). Accordingly, investigation of the phloem translocation function of amino acid transporters (AATs) would contribute to the molecular design of vectorized agrochemicals. A non-phloem-mobile PCA, isolated from the metabolites of the *Pseudomonas* sp. M18 (Su et al., 2010), has been modified, and the resultant L-Val-PCA exhibited the largest phloem mobility among PCA derivatives (Yu et al., 2018). As a model system, castor bean (*Ricinus communis*. L) has been frequently used to test quantitatively the phloem systemicity of pesticides, because simple and reliable tests are available to analyze phloem sap and investigate phloem translocation in seedlings (Kallarackal et al., 1989). A total of 62 AATs were predicted by the bioinformatics analysis after genome sequencing of *R. communis* and RcAAP1 shared high homology with AtAAP3 (Xie et al., 2016). AtAAP3 has high affinity for neutral and basic amino acids. (Fischer et al., 1995). However, the uptake and phloem translocation functions of RcAAP1 toward L-Val-PCA are still unclear.

Yeast (*Saccharomyces cerevisiae*) is a good model for investigating the roles of transporters in uptake of a given amino acid into cells (André, 1995). With this model, amino acid permeases such as AAP1-5 were found from *Arabidopsis thaliana* and functionally identified as transport carriers of neutral amino acids in previous studies (Frommer et al., 1993; Kwart et al., 1993; Fischer et al., 1995; Frommer et al., 1995). The facilitation effects of amino acid transporter-like protein OsATL15 on the thiamethoxam uptake and distribution have been found in *S. cerevisiae* strain W303-1A (Xiao et al., 2022a). In addition, using vacuum agroinfiltration strategy, we constructed an efficient *Agrobacterium*-mediated overexpression method for studying the phloem translocation functions of *Ricinus* genes toward amino acid-pesticide conjugates (Xiao et al., 2022b). With these methods, this study investigated L-Val-PCA uptake in yeast and measured the phloem sap concentration of L-Val-PCA in seedlings by HPLC (high performance liquid chromatography). The relative expression levels of *RcAAP1* after L-Val-PCA treatments of cotyledons, protein structures of RcAAP1 for functional annotation and subcellular localization were also examined.

## Materials and methods

### Plant materials and growths conditions

Seeds of *R. communis* were obtained from the Zibo Academy of Agricultural Science (Shandong, China). Acceleration of seed germination and enhanced growth of seedlings have been described previously (Rocher et al., 2006; Xiao et al., 2022b). Uniform seedlings were selected, and endosperm was carefully removed for further experiments.

### RT-qPCR analysis

To detect the relative expression levels of *RcAAP1*, cotyledons of the endosperm-excised seedlings were incubated in solutions (20 mM MES, 0.25 mM MgCl2, 0.5 mM CaCl2, pH 5.6) containing L-Val-PCA (100 µM), L-Val (100 µM) or PCA (100 µM). A solution containing same 0.5% dimethyl sulfoxide (DMSO) solvent in the L-Val-PCA, L-Val or PCA incubation solutions was set up as control. After incubation for 1 h, 3 h or 6 h, cotyledons were harvested, quickly frozen in liquid nitrogen, and stored at -80°C for RNA isolation. Total RNA from cotyledons was extracted using the E.Z.N.A.^®^ HP Plant RNA Kit (Omega Bio-tek, Norcross, Georgia, USA). The quality of RNA was assessed with an UV-Vis spectrophotometer (Q6000M, Quawell, San Jose, USA) at 260 nm and the ratio A260/A280 ~ 2.0 was used to indicate the purity of the RNA.

Using 1 µg total RNA as template, the first-strand cDNA was synthesized by the HiScript^®^ III 1st Strand cDNA Synthesis Kit (+gDNA wiper) (Vazyme, Nanjing, China). Synthesized cDNA was diluted with nuclease-free H_2_O (1 + 9 v/v) for subsequent qPCR. A set of primers of qRcAAP1-F/R was designed using Primer Premier 5.0 software for qPCR. The qPCR protocol and calculation of the relative expression levels of *RcAAP1* were previously described (Livak and Schmittgen, 2001; Xiao et al., 2022b).

### Yeast expression assay

To determine the effects of RcAAP1 on the uptake of L-Val-PCA, recombinant pYES2-RcAAP1 was constructed as follows. The coding sequence of RcAAP1 was downloaded from the Phytozome database (https://phytozome-next.jgi.doe.gov/). The primers pYES2-RcAAP1-F/R for full-length amplification of RcAAP1 were designed using Primer Premier 5.0 software (Premier Biosoft International, Computing Associates, Palo Alto, USA; Singh et al., 1998) and synthesized by TIANYIHUIYUAN Biotech (Wuhan, China). The 5’ end of the forward primer contained an *Xho* I (NEB, Beijing, China) site and 14 bp homologous sequences, which were located upstream of the *Xho* I site of yeast expression vector pYES2. The 5’ end of the reverse primer also contained an *Xho* I site and 14 bp homologous sequences downstream. A set of universal primers, pYES2-*Xho* I-F/R, were designed for the rapid screening of positive transformants. The forward primer was located upstream of the *Xho* I site of pYES2 at 130 bp, and the reverse primer was located 249 bp downstream of the *Xho* I site.

Phanta^®^ Max Super-Fidelity DNA Polymerase (Vazyme, Nanjing, China) was used for full-length amplification of *RcAAP1* by PCR. Empty vector pYES2 was digested with *Xho* I to obtain the linear plasmid, and then PCR products and linear pYES2 were separated by 1% agarose gel electrophoresis and purified using E.Z.N.A.^®^ Gel Extraction Kit. ClonExpress^®^ II One Step Cloning Kit (Vazyme) was used to connect target fragments to pYES2 by the In-Fusion method (Sleight et al., 2010). The recombinant pYES2-RcAAP1 was introduced into *S. cerevisiae* W303-1A (MATa leu2-3,112 trp1-1 can1-100 ura3-1 ade2-1 his3-11,15) (MiaolingBio, Wuhan, China) using a modified lithium acetate (LiOAc) transformation method (Hill et al., 1991; Yamaki et al., 2017). In the original method, DMSO was added after incubation at 30°C for 45 min. In our method, 10% DMSO was added to the mixture containing 50% polyethylene glycol (PEG-3350), 100 mM LiOAc, 10 mM Tris-HCl and 1 mM EDTA solution (8:1:0.5:0.5) prior to incubation. Transformants were cultured on SD-Glc (Glucose) medium (with agar) for 48-72 h at 30°C. Positive recombinants were screened from the single colonies of SD-Glc agar using PCR and then transferred to SD-Glc medium (without agar) and cultured overnight. Subsequently, 10 μl of cultures was transferred to 100 μl of the new SD-Glc medium and cultured for 4 h. The OD_600nm_ of the cultures was assessed using a dual-beam ultraviolet-visible spectrophotometer (Purkinje General Instrument CO.,LTD, Beijing, China), and cultures were diluted with MilliQ-filtered water to an OD_600nm_ reading of 0.01. Then, 10 μL of each culture was spotted on SD-Gal (2% galactose) medium containing various concentrations of L-Val-PCA (1 mM and 1.5 mM). After incubation for 48-72 h at 30°C, L-Val-PCA sensitive mutants were collected and re-screened using the same protocol. The W303-1A that introduced empty pYES2 was used as control.

To assess L-Val-PCA uptake into the W303-1A, overnight cultures were centrifuged at 2000 g for 5 min and supernatant was discarded (Centrifuge 5430, Eppendorf, Hamburg, Germany). After resuspension by adding 5 mL of fresh SD-Gal, cells were cultured for 3 h at 30°C. Afterwards 5 µL of 50 mM 5-fluorouracil (Solarbio) was added to media and cells were cultured for 1 h to stop cell growth. Cells were diluted with SD-Gal containing 50 μM 5-fluorouracil to a density of 1 x 10^7^ cells per µL. Subsequently, the diluted cells were cultured at 30°C by shaking at 250 rpm for 1 h and centrifuged at 1500 g for 2 min. After the supernatant was discarded, 100 µL of MES-NaOH buffer (0.333 mM MES, 1 mM L-Val-PCA, 50 µM 5-fluorouracil, 2% glucose, pH 5.6) was added to the cultures, which were continuously shaken at 350 rpm for 1 h and centrifuged at 2000 g for 10 min. The supernatant was discarded and the cells were washed five times with ice-cold MilliQ-filtered water. Cells were then resuspended in 1 mL of methanol for 2 min and ultrasonicated for 30 min. After centrifugation of the resuspension at 2000 g for 10 min, the supernatant was obtained for the HPLC (Haineng LC7000, Jinan, China) analysis. The mobile phase consisted of methanol and water containing 0.1% phosphoric acid at a flow rate of 0.8 mL·min^-1^, and the injection volume was 10 μL. Various standard solutions (0.2, 0.3, 0.5, 1, and 2 mg·L^-1^) of the L-Val-PCA for calibration curves were prepared in methanol.

### Bioinformatics analysis

To classify RcAAP1 into protein families, predict domains, model protein structure, and analyze function, the amino acid sequence of RcAAP1 was downloaded from the Phytozome database. Then the sequence was compared to the databases at InterPro (https://www.ebi.ac.uk/interpro/) (Paysan-Lafosse et al., 2023), SMART (http://smart.embl-heidelberg.de/) (Letunic and Bork, 2018; Letunic et al., 2021) and Phyre2 (http://www.sbg.bio.ic.ac.uk/~phyre2/html/page.cgi?id=index) (Kelley et al., 2015). Sequence alignments of amino acids were performed using DNAMAN 8.0 software (Guo et al., 2019).

### Phylogenetic analysis

To find homologous sequences of RcAAP1 and examine molecular evolutionary, the amino acid sequence of RcAAP1 was compared to the NCBI databases (https://www.ncbi.nlm.nih.gov/). Subsequently, multiple sequence alignments in MEGA11 (http://www.megasoftware.net/) were carried out using MUSCLE (Tamura et al., 2021), and trees were constructed by the Neighbor-joining (NJ) method with bootstrap values from 1000 replicates.

### Subcellular localization

To determine the expression and localization of the RcAAP1, plant binary vector pART27-RcAAP1-eGFP was constructed as previously described (Xiao et al., 2022b). Briefly, a set of primers pART27-RcAAP1-F/R were designed by Premier Primer 5.0. The 5’ end of the forward primer contained an *Xho* I site and 14 bp homologous sequences, which were located upstream of the *Xho* I site of pART27-eGFP (College of Agriculture, Yangtze University). The 5’ end of the reverse primer also contained an *Xho* I site and 14 bp homologous sequences downstream. After full-length amplification, plasmid linear of empty pART27-eGFP, 1% agarose gel electrophoresis separation and purification, target fragments were connected to pART27-eGFP by the In-Fusion method. Plant binary vector pCAMBIA1300-35S-PM-mCherry (MiaolingBio) was used as localization marker and pART27-eGFP as control. Subsequently, marker and recombinant pART27-RcAAP1-eGFP were transformed into *Agrobacterium tumefaciens* strain GV3101 (WEIDI, Shanghai, China) by the freeze-melt method, which included freezing for 5 min in liquid nitrogen and thermal shock for 5 min at 37°C (Huang et al., 2021). The transformant carrying recombinants was cultured for 18 h in 5 mL LB medium containing 50 μg/mL spectinomycin (Spec) and 20 μg/mL rifampin (Rif). All antibiotics were from the Solarbio Science & Technology Co., Ltd (Beijing, China). The transformant just carrying the marker was cultured in LB medium containing 50 μg/mL kanamycin (Kana). After centrifugation at 2000 g for 5 min, the collected *Agrobacterium* cells were resuspended in MES solution containing 200 μM acetosyringone (Solarbio), to reach an OD_600nm_ of 0.4. The resuspension carrying the marker was mixed with the resuspension carrying recombinants in 50 mL centrifuge tubes at a ratio of 1:1. Mixtures were placed at room temperature for 1 h, and then transformed into 6-leaf stage *Nicotiana benthamiana* by stab inoculation. After incubation for 72 h at 23 ± 1°C, fluorescence signals were observed at 400 x magnification by laser confocal microscopy (TCS-SP8, Leica, Wetzlar, Germany).

### *Ricinus* seedling expression assay

To demonstrate the expression, phloem localization and phloem translocation functions of RcAAP1 toward L-Val-PCA, recombinant pART27-RcAAP1-eGFP was transformed into seedlings using *Agrobacterium*-mediated overexpression protocol (Xiao et al., 2022b). Briefly, the *Agrobacterium* GV3101 transformant carrying pART27-RcAAP1-eGFP was cultured for 18 h in 400 mL LB medium containing 50 μg/mL Spec, 20 μg/mL Rif. After centrifugation (Avanti JXN-30, Beckman Coulter, California, USA) at 8000 g for 5 min, the *Agrobacterium* cells were collected and resuspended in 200 mL of solution (10 mM MES, 10 mM MgCl_2_ and 200 μM acetosyringone, pH 5.6), to reach an OD_600nm_ of 1.2. Then endosperm-excised seedlings were soaked in the *Agrobacterium* suspension and subjected to vacuum infiltration (JX820D-1, SMAF, Shanghai, China) at 0.09 MPa for 20 plus 20 min (vacuum infiltration for 20 min, then returned to atmospheric pressure, and then infiltrated for another 20 min). When infiltration was complete, seedlings were transferred into Hoagland solution and cultured in a cold-light source incubator (GDX-330, Safu, Ningbo, China) for 72 h at 18°C in the dark (Zhu et al., 2016). Then the seedlings were observed under UV light (UVP BLAK-RAY B-100AP LAMP, Analytic Jena, Jena, Germany) to survey fluorescence signal strength. Cotyledons were observed at 100 x or 400 x magnification and hypocotyls were observed at 200 x magnification by laser confocal microscopy to confirm RcAAP1-eGFP expression. Seedlings with introduced pART27-eGFP were used as controls. Cotyledons cultured for 72 h at 18°C in darkness were harvested, quickly frozen in liquid nitrogen and stored at -80°C for RNA isolation. Total RNA was extracted and the first-strand cDNA was synthesized as above, and the relative expression levels of *RcAAP1* were assessed after overexpression for 72 h. The relative expression levels at 0 h were used as controls. L-Val-PCA phloem sap collection method was similar to that previously described (Rocher et al., 2006). The methods of sample dilution, purification and HPLC analysis followed Xiao et al. (2022b).

### Statistical analysis

Statistical analyses were performed using IBM SPSS Statistics 20 (IBM SPSS Statistics, Chicago, Illinois, USA) for Windows. Significant treatment effects were assessed with ANOVA followed by mean separation using Dunnett’s test at *p* = 0.05. Means and standard deviations were calculated from triplicate measurements.

## Results

### RcAAP1 facilitated the uptake of L-Val-PCA in yeast

**Figure 1:**
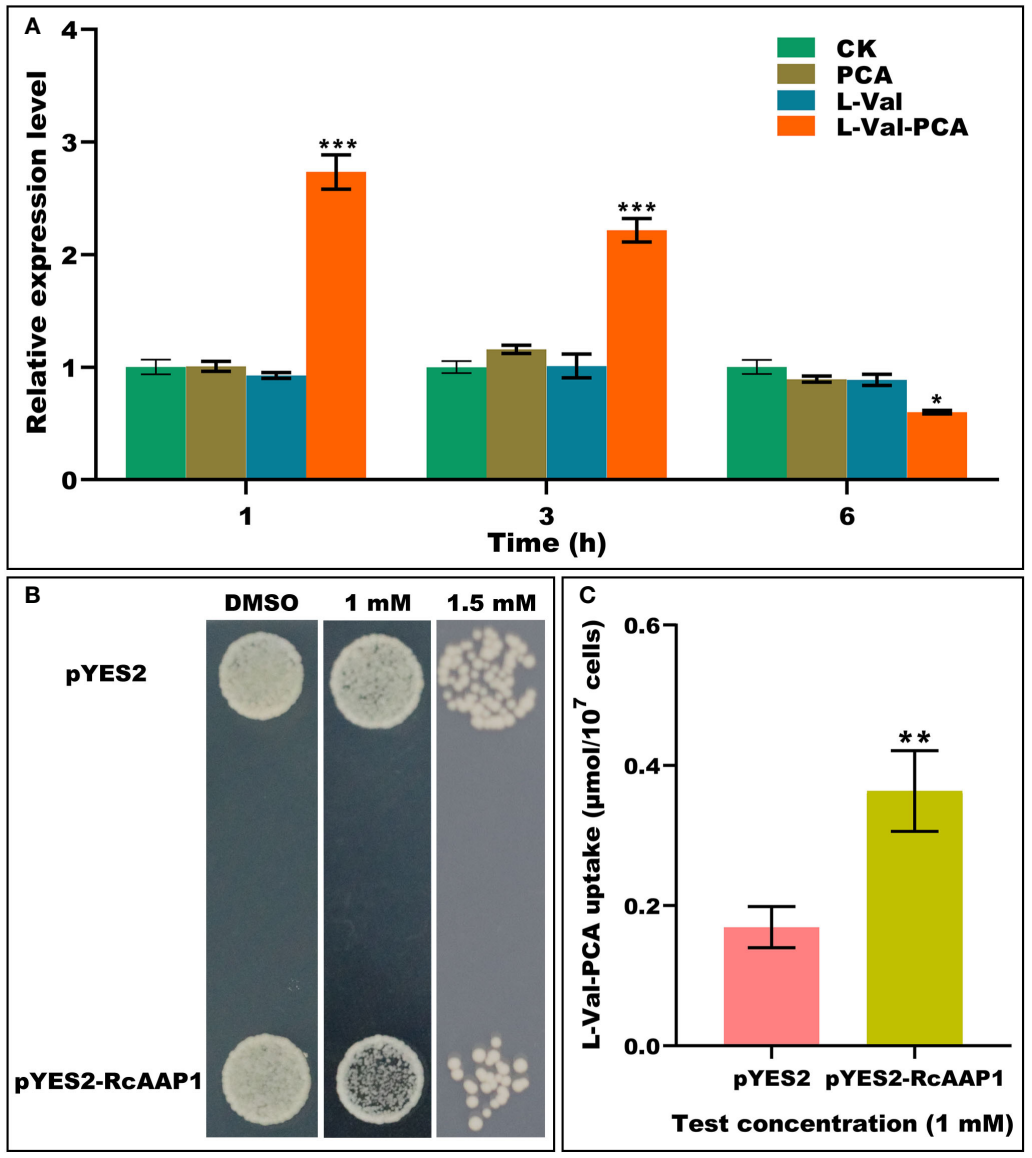
Identification of the effects of RcAAP1 on L-Val-PCA uptake. **(A)** The relative expression levels of *RcAAP1* at 1 h, 3 h, and 6 h after 100 μM PCA, 100 μM L-Val or 100 μM L-Val-PCA treatment of seedling cotyledons. “CK” stands for solvent treatments (0.5% dimethyl sulfoxide, 20 mM MES, 0.25 mM MgCl_2_, 0.5 mM CaCl_2_, pH 5.6). The reference gene *RcActin* was used for normalization of expression. The relative expression level represents fold-change. The experiment used three biological replicates. Asterisks on the graphs indicate statistically significant differences (*P < 0.05, **P < 0.01, ***P < 0.001). **(B)** Growth of *S. cerevisiae* W303-1A carrying pYES2-RcAAP1 with a density of OD_600nm_ = 0.01 on SD-Gal (2% galactose) media containing 1 mM or 1.5 mM L-Val-PCA. The medium containing 0.5% DMSO was used as the control. **(C)** The uptake amount of L-Val-PCA in yeast cells. Transformant carrying empty plasmid pYES2 was set up as the control. The standard curve (y = 0.13052x + 0.72556) was used for HPLC determination of L-Val-PCA. The correlation coefficient was 0.99999. The experiment was replicated three times and different independent samples were used during these biological replicates. Asterisks on the graphs indicate statistically significant differences (*P < 0.05, **P < 0.01, ***P < 0.001).

To investigate the responses of RcAAP1 to PCA, L-Val or L-Val-PCA treatments of castor bean seedlings for various times (1 h, 3 h, and 6 h), the relative expression levels of *RcAAP1* were analyzed by qPCR. As shown in **Figure 1A**, compared to the controls, there were no significant differences in the relative expression levels of *RcAAP1* after PCA and L-Val treatments for 1 h, 3 h, and 6 h. However, *RcAAP1* expression levels were 2.7-fold and 2.2-fold significantly higher than controls at 1 h and 3 h after L-Val-PCA treatment, and 1.7-fold significantly lower than control 6 h after treatment. These results suggested that RcAAP1 responded to the L-Val-PCA treatment, but differed with respect to PCA and L-Val treatments. Then, the effect of RcAAP1 on the L-Val-PCA uptake were surveyed using yeast expression system and the uptake amount of L-Val-PCA was assessed by HPLC analysis. As shown in **Figure 1B**, 1 mM L-Val-PCA treatment slightly inhibited growth of the yeast cells carrying pYES2-RcAAP1 compared to the cells carrying empty pYES2 plasmid at 72 h after transformation, and the 1.5 mM treatment can obviously inhibit the cell growth. However, 0.5% DMSO control had no consistent effect on the growth of the yeast cells carrying either pYES2-RcAAP1 or pYES2. Furthermore, HPLC detection results (**Figure 1C**) indicated that L-Val-PCA amount in the yeast cells carrying pYES2-RcAAP1 (0.36 μmol/10^7^ cells) were 2.1-fold higher than the cells carrying pYES2 (0.17 μmol/10^7^ cells).

### RcAAP1 belongs to the AAP3 family

**Figure 2:**
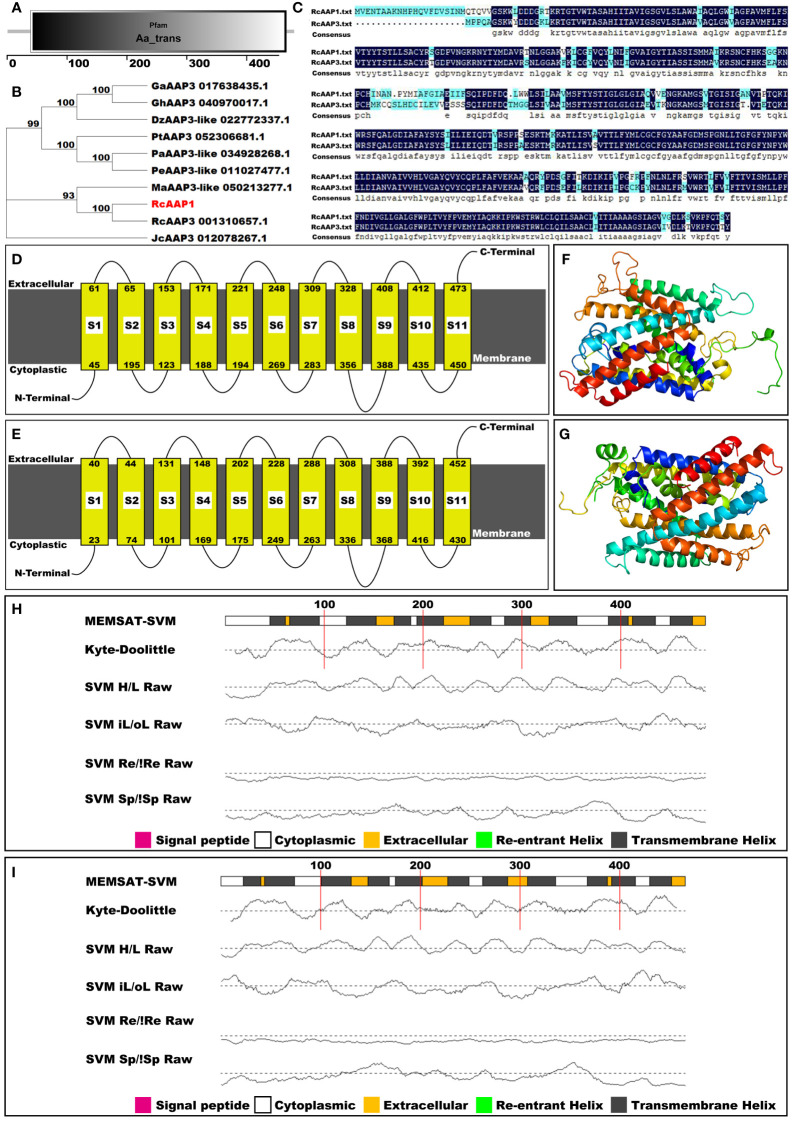
Bioinformatic and phylogenetic analyses of RcAAP1 after submission of amino acid sequence to the databases of interPro, NCBI, SMART, and Phyre2. **(A)** Pfam analysis. **(B)** Phylogenetic analysis of RcAAP1 with homologous AAT sequences from nine species, namely *Durio zibethinus*, *Gossypium arboretum*, *Gossypium hirsutum*, *Jatropha curcas*, *Mercurialis annua*, *Populus alba*, *Populus euphratica*, *Populus trichocarpa*, and *R. communis*. This tree was constructed using MEGA11 with the Neighbor-Joining (NJ) algorithm. The optimal trees were statistically evaluated by bootstrap analysis with 1000 replications and the bootstrap percentages are shown at the appropriate branches. **(C)** Results of sequence alignments of amino acids between RcAAP1 and RcAAP3. **(D, E)** Domain prediction of RcAAP1 and RcAAP3. **(F, G)** Three-dimensional structures of RcAAP1 and RcAAP3. **(H, I)** Secondary structure analysis of RcAAP1 and RcAAP3.

The amino acid sequence of RcAAP1 was compared to the databases at InterPro, NCBI, SMART, and Phyre2 to classify RcAAP1 into protein families, examine molecular evolutionary, predict domains, and model protein structure. Pfam analysis results indicated that RcAAP1 was a member of the amino acid transporter family (**Figure 2A**). However, phylogenetic analysis revealed that RcAAP1 has “highest” homology to AAP3 within nine sequence data set analyzed (**Figure 2B**). Sequence alignment results showed that RcAAP1 had 81% identify with RcAAP3 (**Figure 2C**). Both RcAAP1 and RcAAP3 had a total of 11 domains, and were membrane bound (**Figures 2D, E**). Moreover, **Figures 2F, G** showed the three-dimensional (3D) structures of RcAAP1 and RcAAP3. Phyre2 analysis showed the domains of both RcAAP1 and RcAAP3 had the form of transmembrane helices (**Figures 2H, I**). The protein data bank (PDB) header of RcAAP1 template that rated highest was transport protein, the PDB header of RcAAP3 was membrane protein.

### RcAAP1 was located in the plasma membrane of mesophyll cells and phloem cells

**Figure 3:**
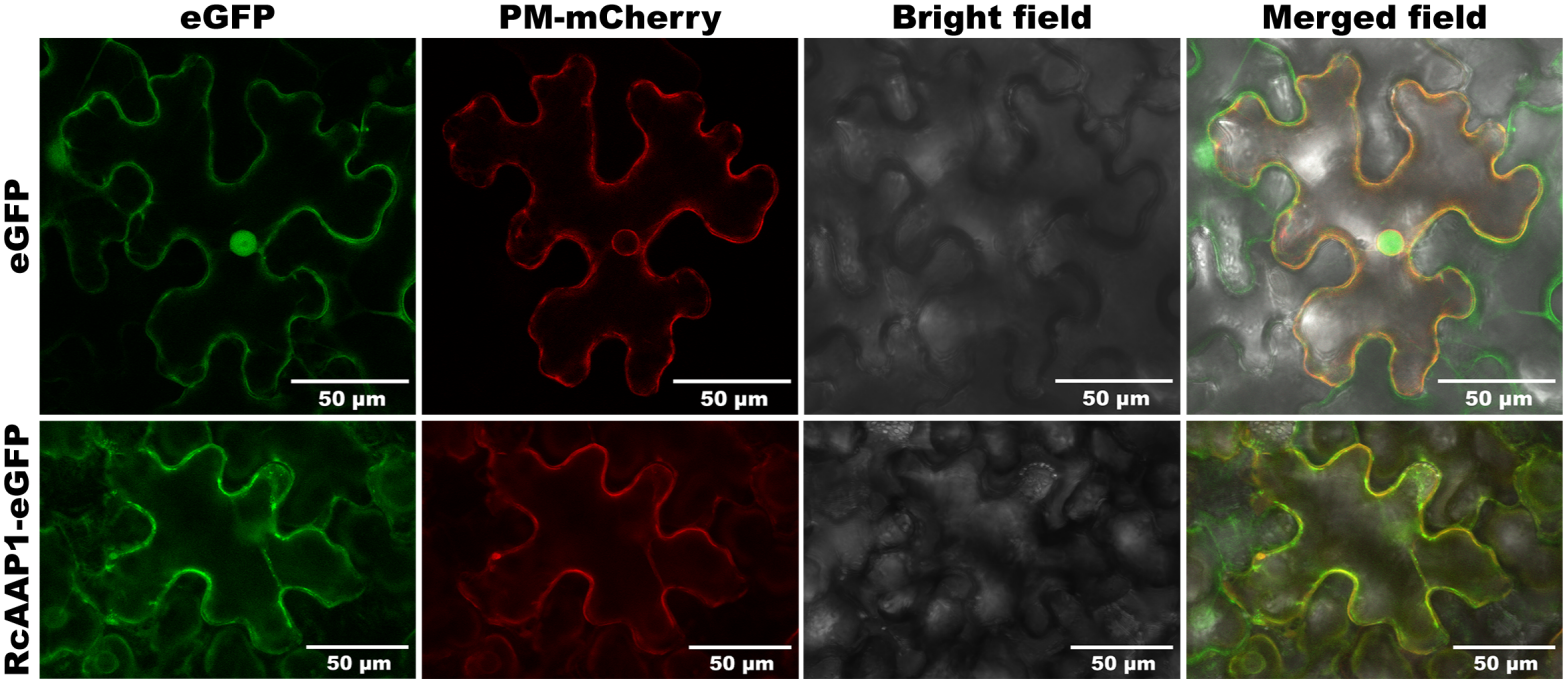
Expression and localization observation of fusion RcAAP1-eGFP in the mesophyll cells of *N. benthamiana* at 400 x magnification by laser confocal microscopy. Plant binary vectors pCAMBIA1300-35S-PM-mCherry and pART27-eGFP was used to transiently expressed DsRed (*Discosoma* red fluorescent protein) and eGFP (enhanced green fluorescent protein), which were used as the localization marker and the control, respectively.

To assess the expression and localization of RcAAP1, plant binary vectors pART27-eGFP and pART27-RcAAP1-eGFP were introduced into *N. benthamiana*. As shown in **Figure 3**, the signals of eGFP in the green fluorescent field were located in the cell nucleus and plasma membrane of *N. benthamiana* at 72 h after transformation. The signals of *Discosoma* red fluorescent protein (DsRed) in the red fluorescent field were located in the nucleus membrane and plasma membrane. The eGFP and DsRed signals were very faintly in the cytoplast. The overlay signals of eGFP and DsRed in merged field were located in the cell nucleus and plasma membrane. For the fusion RcAAP1-eGFP protein, we observed that the green fluorescent signals were located in the plasma membrane of mesophyll cells at 72 h. DsRed signals were also observed in the plasma membrane. The cell shapes of eGFP and RcAAP1-eGFP were unclear under bright field. No fluorescent signals of RcAAP1-eGFP were found in the plasma membrane of epidermal cells.

**Figure 4:**
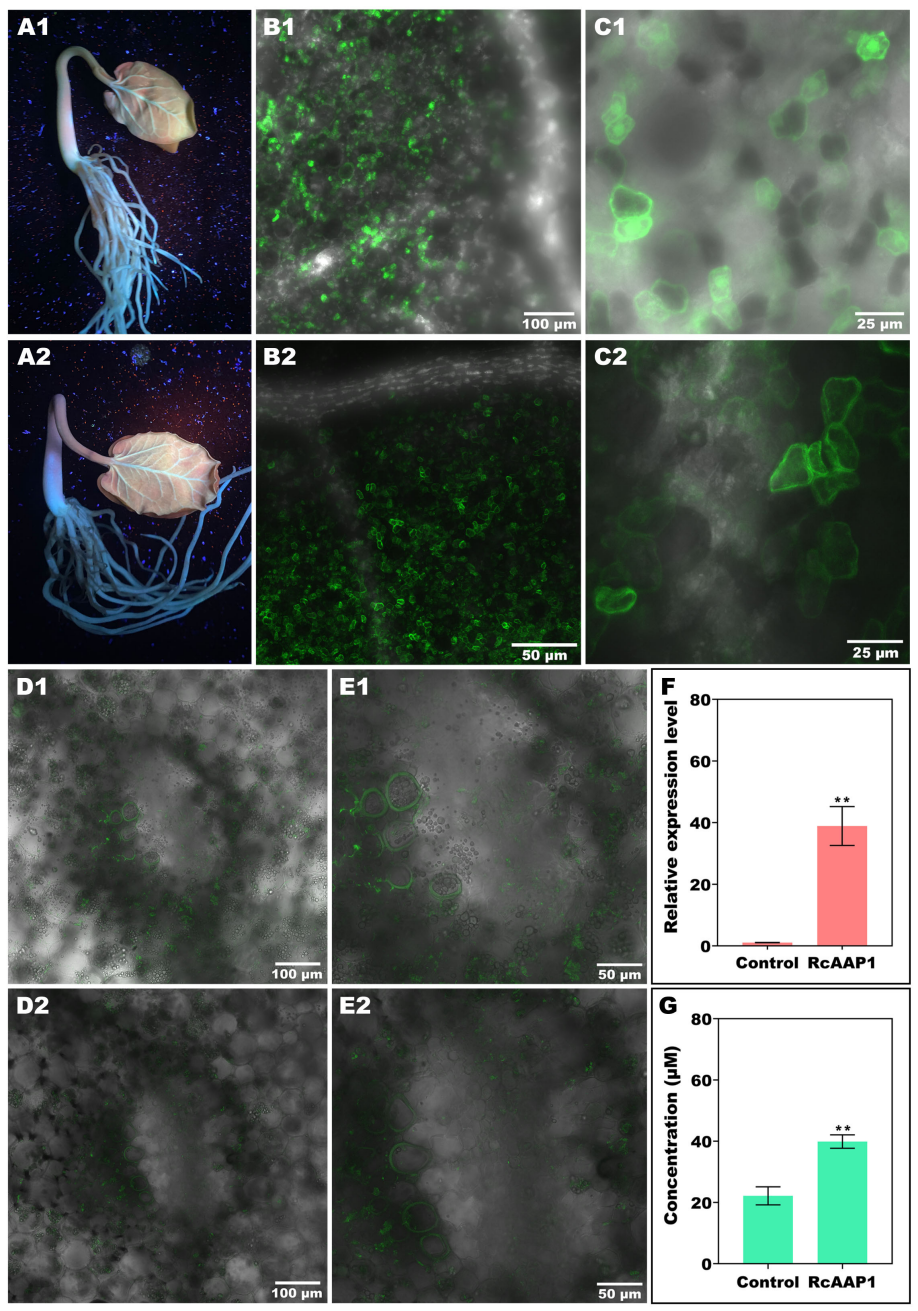
Overexpression of *RcAAP1* were carried out in *Ricinus* seedlings for subcellular localization and phloem translocation functions of RcAAP1 toward L-Val-PCA. **(A1, A2)** Confirm of the expression of eGFP and fusion RcAAP1-eGFP under UV light at 72 h after transformation of empty vector pART27-eGFP and recombinants pART27-RcAAP1-eGFP into *Ricinus* seedlings by vacuum agroinfiltration. **(B1, B2)** Cotyledon observation of eGFP and RcAAP1-eGFP at 100 x magnification by laser confocal microscopy. **(C1, C2)** Cotyledon observation of eGFP and RcAAP1-eGFP at 400 x magnification. **(D1, D2)** Phloem observation of eGFP and RcAAP1-eGFP at 200 x magnification by laser confocal microscopy. **(E1, E2)** The enlargements of phloem cells that overexpressed eGFP gene and RcAAP1 gene, respectively. **(F)** qPCR analysis of the relative expression levels of *RcAAP1* after overexpression of *RcAAP1* for 72 h. **(G)** HPLC detection of the phloem sap concentrations of L-Val-PCA after overexpression of *RcAAP1* for 72 h. The experiment was replicated three times and different independent samples were used during these biological replicates. Different asterisks on the graphs indicate statistically significant differences (**P < 0.01).

The expression and localization of RcAAP1 was also observed in *R. communis* seedlings after transformation of these two vectors pART27-eGFP and pART27-RcAAP1-eGFP by vacuum agroinfiltration. Green fluorescent signals of the eGFP and fusion RcAAP1-eGFP proteins were observed around the leaf vein and basal part of stem at 72 h after transformation (**Figures 4A1, A2**). Subcellular observation of cotyledon at 100 x magnification under merged field by laser confocal microscopy showed that both eGFP signals and RcAAP1-eGFP signals were located in the mesophyll cells but no signals in the leaf vein (**Figures 4B1, B2**). The cotyledon observation at 400 x magnification under merged field showed that eGFP signals were located in the plasma membrane, cytoplasm, and nucleus (**Figure 4C1**). but RcAAP1-eGFP signals were in the plasma membrane and cytoplasm (**Figure 4C2**). Furthermore, subcellular observation of hypocotyls at 200 x magnification under merged field showed that both eGFP signals and RcAAP1-eGFP signals were located in the plasma membrane of phloem cells (**Figures 4D1,D2**). **Figures 4E1,E2** showed that the enlargements of phloem cells that overexpressed eGFP gene and RcAAP1 gene, respectively.

### Overexpression of *RcAAP1* increased the phloem mobility of L-Val-PCA in *Ricinus* seedlings

To investigate the effect of RcAAP1 on the phloem translocation of L-Val-PCA, these two vectors pART27-eGFP and pART27-RcAAP1-eGFP were transformed into seedlings by vacuum agroinfiltration. qPCR analysis indicated that the relative levels of RcAAP1 were 36-fold more than the control (**Figure 4F**). As shown in **Figure 4G**, HPLC detection results revealed that the phloem sap concentrations of L-Val-PCA (39.88 μM) after overexpression of *RcAAP1* for 72 h were 1.8-fold higher than the control (22.15 μM).

## Discussion

Amino acid-agrochemical conjugates can use endogenous amino acid carriers to acquire phloem mobility (Chollet et al., 1997). A large number of amino acid-agrochemical conjugates such as insecticidal fipronil derivatives, chlorantraniliprole derivatives, fungicidal fenpiclonil, and PCA derivatives were designed and synthesized for improvement of the phloem mobility of parent ingredients (Chollet et al., 2004; Chollet et al., 2005; Wu et al., 2012; Niu et al., 2016; Wu et al., 2016; Niu et al., 2017; Yao et al., 2017; Yu et al., 2018). To investigate whether RcAAP1 was involved in the uptake and phloem translocation of L-Val-PCA, the relative expression levels of *RcAAP1* were analyzed after L-Val-PCA treatment. L-Val treatment was set up as the positive control because we assumed that RcAAP1 enabled transport of valine groups of L-Val-PCA (Marvier et al., 1998). Because PCA is not phloem mobile, we assumed that PCA treatment had no induction effect on expression of RcAAP1, and used this treatment as the negative control (Niu et al., 2016; Yu et al., 2018). We found that *RcAAP1* expression levels showed no significant differences compare to the control after PCA and L-Val treatment for 1 h and 3 h, but, in contrast, were up-regulated after L-Val-PCA treatment. RcAAP1 lacked responses to the L-Val treatment, suggesting that L-Val-PCA acquiring cell uptake and phloem translocation may not rely on recognition effect of RcAAP1 on the valine groups of the conjugates. The silence of RcAAP1 to L-Val treatment may be due to its low affinity for L-Val. RcAAP1 is a neutral and basic amino acid transporter with a wide substrate specificity, while valine (and other amino acids such as threonine, isoleucine, leucine, phenylalanine, tyrosine, and tryptophan) show low inhibition effects on histidine uptake after expression of *RcAAP1* (Marvier et al., 1998). In this study, RcAAP1 was found 81% identify with the RcAAP3, which was ever expressed in *S. cerevisiae* 2512c (*Mat-a*, *gap1*) with *ura*3-52 mutation and valine exhibited a large inhibition effect on the [^14^C]citrulline uptake (Neelam et al., 1999). Moreover, the direct uptake rate of radiolabeled L-valine into *S. cerevisiae* 22574d was relatively low after expression of *AtAAP3* (Fischer et al., 1995). These results of inhibition uptake may account for the lack of response of RcAAP1 to L-Val treatment, though RcAAP1 has 79% protein identity with AtAAP3 (Xie et al., 2016).

Uptake of glyphosate and paraquat has been found carrier-mediated by the transporters of phosphate and polyamine, respectively (Hart et al., 1992; Denis and Delrot, 1993), though these two herbicides are not vectorized agrochemicals. Salicylic acid and thiamethoxam also involve a carrier-mediated transport system (Rocher et al., 2009; Xiao et al., 2022a), suggests that a number of phloem-mobile agrochemicals themselves can be recognized and transported by the according plant transporters. Therefore, changes of steric configuration of L-Val-PCA compared to PCA may bring about the response of RcAAP1 to the L-Val-PCA treatment (Zhu et al., 2019). The expression levels of *RcAAP1* still showed no significant differences compare to the control after PCA and L-Val treatment for 6 h, however, were down-regulated after L-Val-PCA treatment. The reason why a reduction in the expression levels at 6 h after Val-PCA treatment may be related to an uptake plateau appearing in the phloem sap concentration of L-Val-PCA. Rocher et al. (2006) found that salicylic acid levels collected from phloem sap reached a plateau from 2-4 h after the start of *Ricinus* cotyledons exposure to incubation solution.

This study found that RcAAP1 had 81% identify with a previous RcAAP3 (Neelam et al., 1999). SMART and Phyre2 analyses showed that both RcAAP1 and RcAAP3 had 11 domains and RcAAP1 was similar to RcAAP3 regarding the 3D structures. However, the PDB template of RcAAP1 was a transport protein, the RcAAP3 template was a membrane protein. That suggests the transport functions of RcAAP1 may be different from the RcAAP3 functions especially in the aspect of phloem translocation. Therefore, the binding sites of L-Val-PCA with various AATs will be checked in next work.

Amino acid transporter superfamily (ATF) mainly includes five subfamilies: AAPs, ProTs (proline transporters), LHTs (lysine histidine transporters), ANTs (aromatic and neutral amino acid transporters) and AUXs (auxin permeases) (Fischer et al., 1998; Ortiz-Lopez et al., 2000; Tegeder and Ward, 2012). The membranes of ATF contain 9-11 putative membrane-spanning domains with cytosolic N and extracellular C termini (Wipf et al., 2002). Pfam analysis in this study confirms RcAAP1 with 11 transmembrane domains. Furthermore, the toxic actions of 1 mM L-Val-PCA resulted in slight inhibition of the growth of yeast cells after transformation of pYES2-RcAAP1 into *S. cerevisiae* W303-1A and 1.5 mM L-Val-PCA treatment exhibited obvious inhibition. Those results imply that RcAAP1 increases the uptake of L-Val-PCA across plasma membrane. As well, the activity of the transmembrane domains should be examined more closely to perhaps further increase translocation of pesticides.

This study confirms that RcAAP1 is a membrane protein and locates in the plasma membrane of mesophyll cells by subcellular localization in *N. benthamiana*. Vacuum infiltration of *Agrobacterium* for transient gene expression or post-transcriptional gene silencing in intact plant is a rapid, scalable, and useful method without the need to generate transgenic plants. (Arzola et al., 2011; Shamloul et al., 2014; Kaur et al., 2021). We previously applied this method in *Ricinus* system for the phloem translocation functions of RcLHT1 and RcLHT7 toward L-Val-PCA (Xiao et al., 2022b). The *Ricinus* system was also used in this study for the localization and phloem translocation functions of RcAAP1. The subcellular observation under merged field visualization showed that RcAAP1-eGFP signals were not only located in the plasma membrane of mesophyll cells bur also in the plasma membrane of phloem cells at 72 h after transformation of vector pART27-RcAAP1-eGFP into *Ricinus* seedlings. Moreover, a standard curve (y = 0.13052x + 0.72556) was built in this study for calculation of L-Val-PCA uptake concentration. The correlation coefficient was 0.99999. The phloem sap concentration of L-Val-PCA was significantly improved after overexpression of *RcAAP1* for 72 h. These results suggest that RcAAP1 in the plasma membrane can recognize L-Val-PCA and load it into *Ricinus* sieve tubes for phloem translocation. Apart from that, green fluorescent signals of fusion RcAAP1-eGFP are observed in the plasma membrane of mesophyll cells and phloem cells of seedlings suggests that the *Ricinus* system has potential application in the subcellular localization of foreign proteins.

In conclusion, plasma membrane-located RcAAP1 is a member of amino acid permease AAP3 family. In its role as a transport carrier, it can not only facilitate the uptake of L-Val-PCA but also load L-Val-PCA into *Ricinus* sieve tubes for phloem translocation.

## Data availability statement

The original contributions presented in the study are included in the article/**Supplementary Materials**. Further inquiries can be directed to the corresponding author.

## Author contributions

JL designed the study. YX and CH performed the experiments. CH analyzed the data. YX wrote this paper, and TH revised it. All authors contributed to the article and approved the submitted version.
