# Supplementary material for: Amino acid permease RcAAP1 increases the uptake and phloem translocation of an L-valine-phenazine-1-carboxylic acid conjugate
Source: Front Plant Sci. 2023 Jun 2;14:1191250. doi: 10.3389/fpls.2023.1191250 (PMC10272580; doi:10.3389/fpls.2023.1191250)
Supplement: Supplementary file 1 [file DataSheet_1.docx]

Supplementary Material

Amino acid permease RcAAP1 increases the uptake and phloem translocation of an L-valine-phenazine-1-carboxylic acid conjugate

**Yongxin Xiao*****, Ciyin Hu*****, Tom Hsiang，Junkai Li**

*** Correspondence:** Junkai Li: junkaili@sina.com

# Supplementary Data

### Pfam analysis results: <https://www.ebi.ac.uk:443/interpro//result/InterProScan/iprscan5-R20230318-171846-0983-54744595-p1m/>

### SMART analysis results: <http://smart.embl-heidelberg.de/smart/job_status.pl?jobid=2212332449269711679638854JmgtClFyFc>, domains within *Ricinus communis* protein O82044_RICCO (O82044).

# Supplementary Figures

**
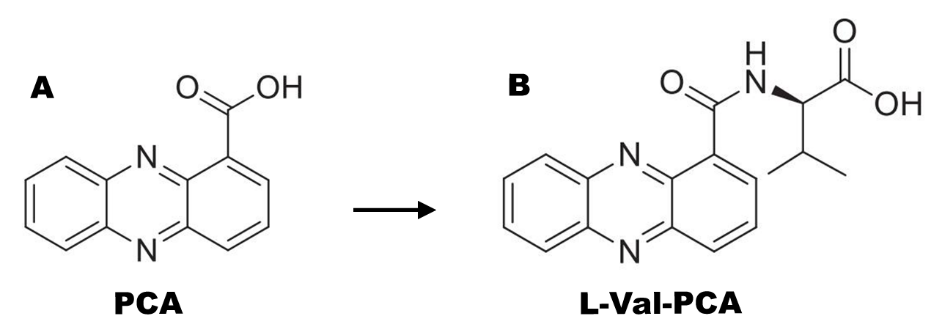
**

**Supplementary Figure 1.** The structures of the L-Val-PCA (L-valine-phenazine-1-carboxylic acid conjugate) and PCA.


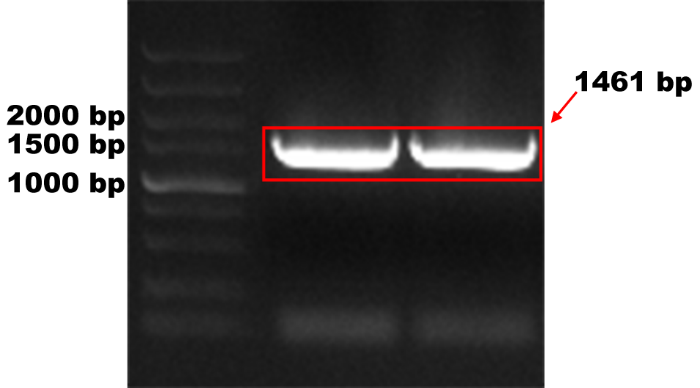


**Supplementary Figure 2.** Full-length amplification of RcAAP1 gene.


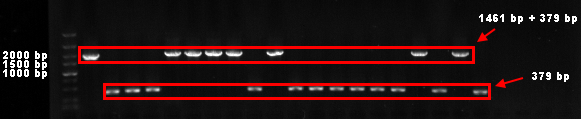


**Supplementary Figure 3.** Rapid screening of positive transformants pYES2-RcAAP1 in *Escherichia coli* DH5α by PCR.


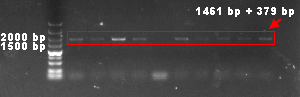


**Supplementary Figure 4.** Rapid screening of positive transformants pYES2-RcAAP1 in yeast W303a by PCR.


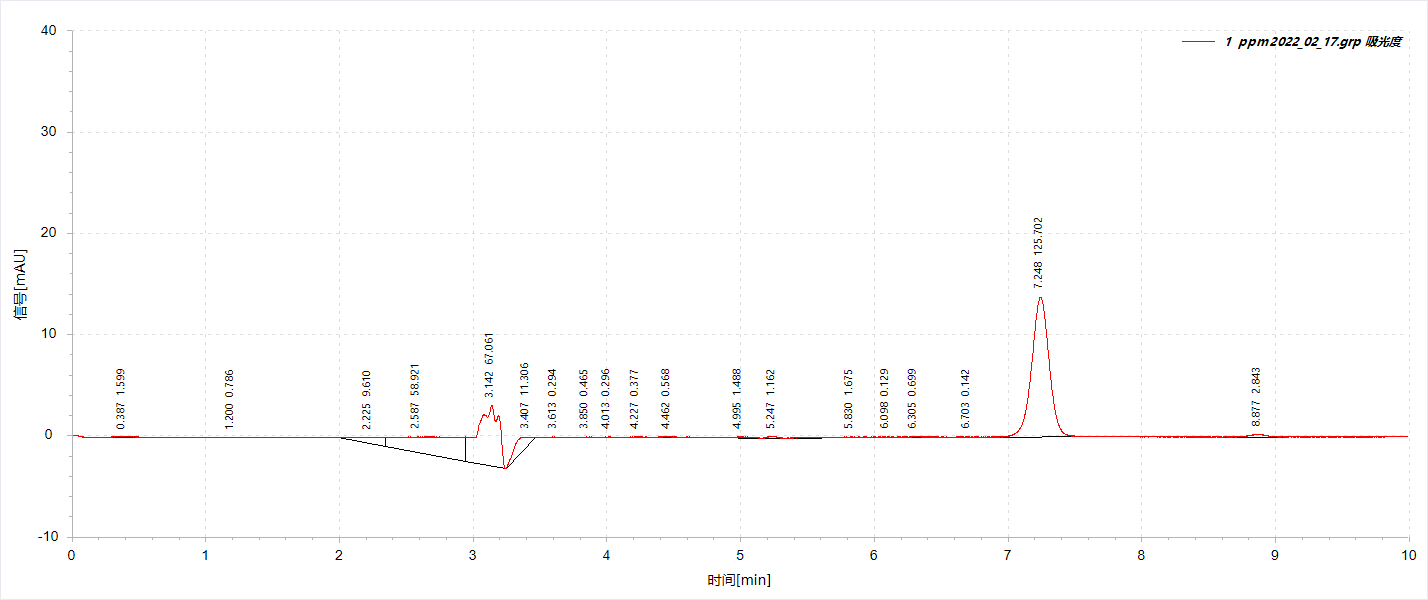


**Supplementary Figure 5.** HPLC detection of a standard solution (1 mg·L^-1^) of L-Val-PCA, the absorption peak of standard L-Val-PCA appeared at 7.248 s.


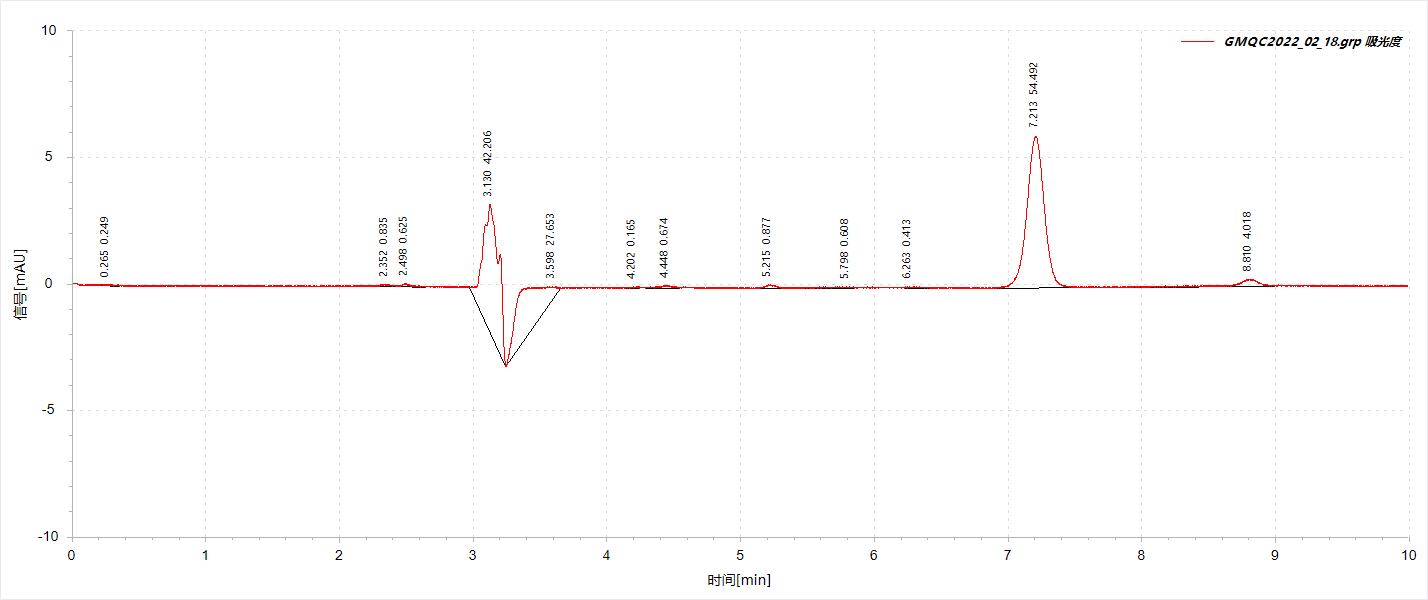


**Supplementary Figure 6.** HPLC detection of L-Val-PCA in *Saccharomyces cerevisiae* W303a after expression of *RcAAP1* for 16 h, the absorption peak of standard L-Val-PCA appeared at 7.217 s.


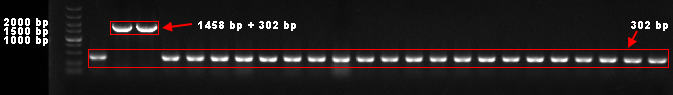


**Supplementary Figure 7.** Rapid screening of positive transformants Part27-RcAAP1-eGFP in *Escherichia coli* DH5α by PCR.


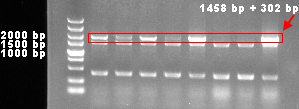


**Supplementary Figure 8.** Rapid screening of positive transformants pART27-RcAAP1-eGFP in *Agrobacterium tumefaciens* GV3101 by PCR.


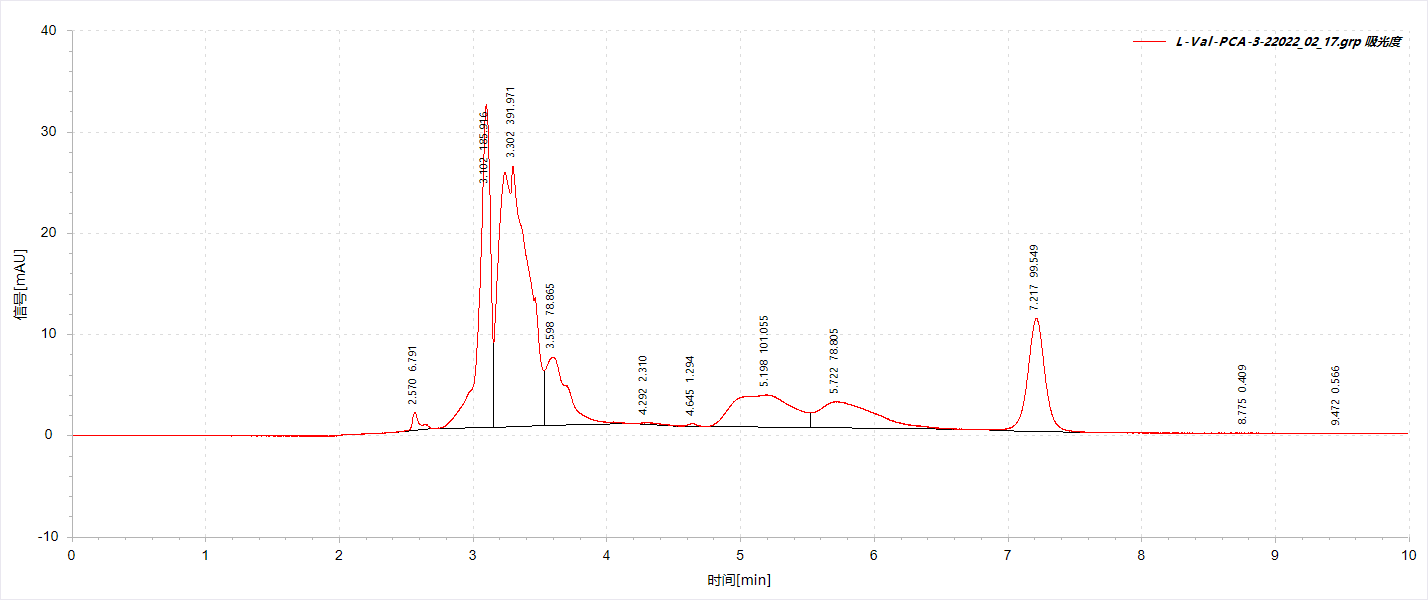


**Supplementary Figure 9.** HPLC detection of L-Val-PCA in the phloem sap of *Ricinus* seedlings after overexpression of *RcAAP1* for 72 h. The L-Val-PCA solution has been diluted 10-fold. The absorption peak of standard L-Val-PCA appeared at 7.217 s.


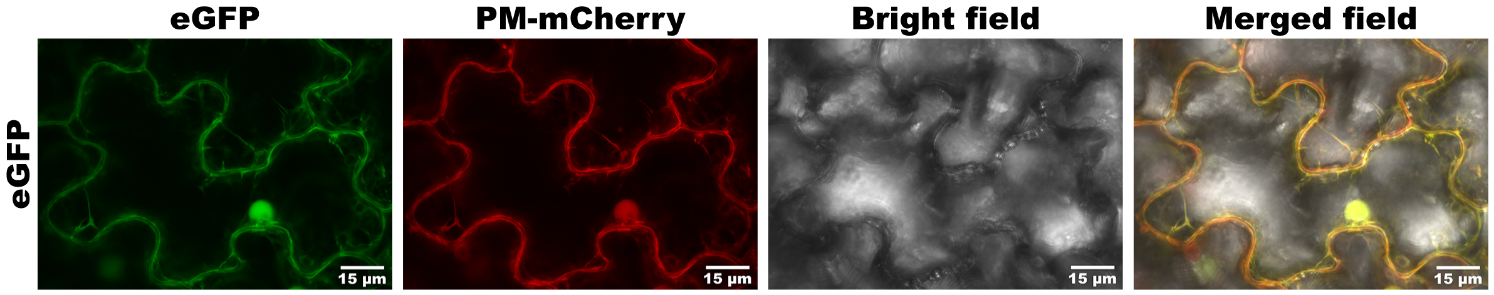


**Supplementary Figure 10.** Expression and localization observation of eGFP in the mesophyll cells of *Nicotiana benthamiana* at 400 x magnification by laser confocal microscopy. Plant binary vectors pCAMBIA1300-35S-PM-mCherry was used to transiently expressed DsRed (*Discosoma* red fluorescent protein), which were used as the localization marker (Xiao and Wei et al, unpublished).

**TABLE S1 The primers used for qPCR, full-length amplification, subcellular localization, and overexpression.**

| Seq. No. | Name | Primer sequence (5’-3’) | Tm (°C) | Product (bp) | Purpose | Reference |
| --- | --- | --- | --- | --- | --- | --- |
| 1 | qRcAAP1-F | TATCTGCCTGCTACCGTTCTG | 58.2 | 150 | qPCR | This study |
|  | qRcAAP1-R | TGTATCCAATGGCAACTCCG | 59.1 |  |  |  |
| 2 | RcActin-F | GTGCTTGATTCTGGTGATGGC | 60.2 | 239 | qPCR | Mao et al., 2017 |
|  | RcActin-R | TTGGCAGTCTCAAGTTCTTGCT | 59.4 |  |  |  |
| 3 | pYES2-RcAAP1-F | CACACTGGCGGCCGCTCGAGATGGTTGAGAACACAGCAGCC | 59.9 | 1461 | Full-length amplification of RcAAP1 for Yeast uptake | This study |
|  | pYES2-RcAAP1-R | CCTCTAGATGCATGCTCGAGTCAGTAAGAGGTCTGGAATGGCT | 59.9 |  |  |  |
| 4 | pYES2-*Xho* I-F | CGGATCGGACTACTAGCAGCT | 59.0 | 385 | Rapid PCR screening of positive recombinants | This study |
|  | pYES2-*Xho* I-R | CGTCCCAAAACCTTCTCAAGC | 61.4 |  |  |  |
| 5 | pART27-RcAAP1-F | TTTGGAGAGGACACGCTCGAGATGGTTGAGAACACAGCAGCC | 59.9 | 1458 | Full-length amplification RcAAP1 for subcellular localization and overexpression | This study |
|  | pART27-RcAAP1-R | GCCCTTGCTCACCATCTCGAGGTAAGAGGTCTGGAATGGCTTGA | 60.3 |  |  |  |

# The restriction enzymes marked in yellow. The homologous sequences located at the upstream or downstream of restriction enzymes for In-Fusion are marked in other colors.
